# Clinical characterization of a novel episodic ataxia in young working Cocker Spaniels

**DOI:** 10.1111/jvim.17268

**Published:** 2024-12-23

**Authors:** Clara Sarró, Catherine Stalin, Rodrigo Gutierrez‐Quintana, Ana Cloquell

**Affiliations:** ^1^ Small Animal Hospital, School of Veterinary Medicine University of Glasgow Glasgow United Kingdom; ^2^ Moorview Referrals Cramlington United Kingdom

**Keywords:** body swaying, cerebellar, paroxysmal movement disorder, titubation

## Abstract

**Background:**

Episodic ataxias (EAs) are a rare group of paroxysmal movement disorders (PMD) described in human medicine with only one suspected case described in veterinary literature.

**Hypothesis/Objectives:**

This study aimed to provide clinical description of a suspected primary EA in working Cocker Spaniel (WCS) dogs.

**Animals:**

Seven WCS dogs with suspected primary EA.

**Methods:**

Descriptive, retrospecitve, multicenter study. Clinical signs, video footage, investigations, treatment, and outcome were reviewed. Owners of affected dogs were invited to complete a questionnaire.

**Results:**

The mean age at clinical onset was 4 months. Signs were acute and included episodic body swaying, titubation, cerebellar ataxia, wide‐base stance, and hypermetria, all while mentation remained unaltered. Neither autonomic nor vestibular signs nor hyperkinetic movements were observed. Duration of episodes ranged from 30 minutes up to 24 hours, and their frequency varied from weekly to once every 5 months. When investigations were performed, results revealed no abnormalities except for 1 dog that had increased gluten antibody titers. None of the dogs deteriorated, and in dogs with available follow‐up (5/7) the frequency of episodes decreased or completely resolved, from which the majority (4/5) received gluten‐free diet.

**Conclusion and Clinical Importance:**

A novel PMD was identified in young WCS, manifesting as EA. The condition is suspected to have a primary (genetic) etiology, although the cause of this manifestation has not yet been identified. Episodic ataxia in our WCS had a good prognosis. Veterinarians must be aware of this presentation, and further investigations are needed to determine the origin of the clinical signs.

**Abbreviations**
CSF: cerebrospinal fluid
EAs: episodic ataxias
EEG: electroencephalogram
FLAIR: fluid attenuation inversion recovery
MRI: magnetic resonance imaging
PMDs: paroxysmal movement disorders
PxDs: paroxysmal dyskinesias
WCS: working Cocker Spaniels

## 1 INTRODUCTION

The expanding spectrum of paroxysmal movement disorders (PMDs) in small animals comprises a group of neurological conditions manifesting as episodic, abnormal, self‐limiting, and involuntary movements.^1^, ^2^ Those movements include tremors, peripheral nerve or muscle hyperexcitability (fasciculations, myokymia, neuromyotonia, myotonia, cramps, tetanus/tetany), myoclonus, paroxysmal dyskinesia (PxD), and dystonia.^1^ Usually, autonomic signs are absent, there is no loss of consciousness and neurological examination is normal between episodes.^2^ Clinical signs can last seconds, minutes or hours.^1^ Given the episodic nature of signs, video evaluation is useful to identify features displayed during the event, which further aid in distinguishing PMDs from other conditions.^3^

Similarly, humans also develop PMDs which are classified in 2 main categories based on phenomenology: paroxysmal dyskinesias (PxDs) and episodic ataxias (EAs). While PxDs are characterized by transient episodes of hyperkinetic movements, EAs are rare disorders characterized by recurrent attacks of cerebellar dysfunction.^4^ Main signs include truncal ataxia, incoordination, and vertigo.^4^, ^5^ Episodic ataxias are primary (inherited) or secondary (acquired).^4^ Primary EA is caused by different subtypes of genetic mutations (at least 8 subtypes—EA1‐8 have been described).^5^ Most primary forms occur as sporadic or familial cases with autosomal dominant inheritance, and onset of manifestations is often set in childhood or adolescence.^4^ Recurrence is variable ranging from a few episodes per year to several per day.^4^ Secondary causes of EA usually begin after the second decade of life, and are caused by different disorders such as structural, immunological, metabolic or neurodegenerative disorders.^4^ In the veterinary literature, only a single case of a suspected primary EA has been reported previously (14 years ago) in a 4‐year‐old Bichon Frise.^6^

The objective of this study was to provide precise clinical characterization and describe long‐term outcomes in 7 unrelated young working Cocker Spaniel (WCS) exhibiting EA. This condition has not been reported in multiple individuals of the same breed. These initial findings are intended to draw the attention of clinicians and raise awareness of this clinical presentation.

## 2 MATERIALS AND METHODS

Ethical approval was granted by the Research Ethics Committee of the School of Veterinary Medicine of the University of Glasgow (EA42/22).

Seven young WCS were identified in the medical records of the University of Glasgow Small Animal Hospital and Moorview Referrals between January 2021 and May 2023 which were presented for evaluation of recurrent paroxysmal episodes of incoordination. A retrospective review of their clinical records was performed. Their neurological examination and video footage of the abnormal episodes was available in all cases and reviewed by all the authors independently. All the videos were evaluated carefully to assess mentation, posture, gait, autonomic signs, presence of vestibular signs or presence of other movement disorders (dyskinesia, myotonia, dystonia, etc.).

Medical records were evaluated including signalment, history, presenting complaint, physical and neurological examination, and abnormalities from video recording of the episodes. Additionally, blood analysis (CBC, biochemistry, and electrolytes), magnetic resonance imaging (MRI) findings, cerebrospinal fluid (CSF) analysis, cardiac scan, specific blood tests (including gliadin IgG and tissue transglutaminase 2 IgA − tTg‐IgA), treatments, and outcome were evaluated when available.

All owners were invited to complete a specifically designed questionnaire (included in Supporting Information). Questions were aimed at acquiring detailed information about the characterization of the episodes, pre‐ and post‐ictal signs identification, triggers detected, and progression of each dog. Questions were also performed to evaluate the presence of other comorbidities. Descriptive statistics were performed and median and range are presented.

## 3 RESULTS

### 3.1 Study cohort

Seven WCS dogs were identified with a history of at least 2 episodes of incoordination and body swaying. Two dogs were male and 5 were female, of which 2 were neutered. The median age of onset was 4 months (ranged from 3 to 5 months). All dogs were sourced from different breeders within the UK, and no relationships were identified among them. In one case, a littermate of one of the dogs had experienced “epilepsy‐like” episodes, but further information was unavailable. No previous history of other disorders was reported in any of the cases. Further details of each dog are summarized in the Supporting Information.

### 3.2 Characterization of episodes

Video evidence was provided for all 7 cases, exhibiting common features in all dogs which included cerebellar ataxia, hypermetria in the thoracic limbs, wide base stance (Video S1), head titubation (Video S2) and body swaying from side to side and occasionally cranially and caudally (Videos S3 and S4). Dogs appeared to be conscious with intact mentation (Videos [Link], [Link]). No vestibular signs were observed. Only 1 dog (Case 3) experienced intermittent, fine, rapid head or body postural tremors, or a combination or both, while lying down during 1 episode presumed to be stress‐related tremors.^7^ However, no other hyperkinetic movements (dyskinesia, myotonia, dystonia, etc.) were identified. Three dogs (3/7) exhibited mild weakness observed as mild instability of the limbs that bend or knuckle spontaneously at the level of the carpal region (Video S5).

### 3.3 Questionnaire data

Five owners completed an interview questionnaire. All owners described episodes as an acute onset of head and truncal sway or bobbing, along with a “drunken gait” (later characterized as cerebellar ataxia from video footage). All owners believed their dog to be conscious during the episode. However, dogs were described as quiet (5/5), less responsive to their call (5/5), subdued (1/5) or confused (1/5). Four owners (4/5) recognized signs of aura, described as an abnormal period (usually minutes) of dullness or lethargy. In just 1 dog (1/5), the aura was characterized by either quietness or excessive excitement, referred to as “zoomies.” Additionally, 3 dogs (3/5) were reported to exhibit a kyphotic or arched posture (not always displayed in video footage). Urination was anecdotally reported in 2 cases, occurring exclusively during the initial episodes. None of the owners reported signs of stiffness or rigidity during episodes.

The duration of these episodes ranged from 30 minutes up to 24 hours. The frequency of the episodes varied, with some dogs experiencing 1 episode a week and another (Case 4) having no more than 2 episodes during the follow up (18 months). Moreover, episodes could manifest at any time during the day, except for 1 dog where they were exclusively witnessed in the evening. For that dog, the owners worked away from home meaning episodes could have been missed during their absence.

Four (4/5) owners reported possible triggers for the episodes including extraneous exercise such as a long hike, a walk on the beach or swimming in the sea as well as long‐lasting excitement such as a family gathering or a car journey. Four owners (4/5) reported their dog having learned to adapt to the episodes by seeking a place to rest (usually their bed) in order to sleep through the event. Four dogs were not interested in drinking or eating during episodes, while 1 dog was described to have trouble reaching the bowl during episodes. Signs of lethargy or exhaustion were described by 3 out of 5 (3/5) owners in the post‐ictal phase, as dogs tended to sleep throughout the next day after an episode, which was unlike their usual puppy‐like behavior.

Moreover, given the extended duration of signs, which could persist for several hours to an entire day, it was documented that 4 (4/5) dogs experienced increased thirst and hunger during the subsequent post‐ictal phase. All dogs were back to their normal self within 48 hours of episode termination. Dogs remained normal between episodes. Based on owner's observation, the episodes were not believed to have significantly affected the overall quality of life in these dogs.

### 3.4 Diagnostic evaluation

Physical and neurological examinations, CBC, and serum biochemistry profile (including electrolytes) did not reveal abnormalities in all 7 dogs. Brain MRI and CSF analysis results were normal in 3 dogs (3/7).

Further diagnostics included comprehensive urinalysis (3/7), organic acidurias (3/7), ammonia (3/7), bile acid stimulation test (2/7), ionized calcium (2/7), cobalamin and folate (2/7), *Toxoplasma* and *Neospora* antibodies titers (3/7), L‐carnitine serum levels (2/7), and modified gliadin peptide immunoglobulin G (gliadin IgG) and tTg‐IgA levels (2/7). All those investigations were normal except from 1 dog (Case 1) that tested positive for gluten sensitivity and another dog (Case 4) that had lower cobalamin levels (251 pg/mL, RI: >275 pg/mL). Moreover, a complete blood work was taken from Case 1 during an episode (blood glucose, ammonia, pre‐bile acids, lactate, urinalysis, and organic acidurias) which resulted normal.

### 3.5 Medical treatment

Medical treatment options were discussed on a case‐by‐case basis. Only 1 owner (Case 1) opted to trial antiepileptic medication because of the frequency and duration of the episodes (4 episodes per month). Oral levetiracetam (30 mg/kg q8h) and phenobarbital (2.5 mg/kg q12h) were started. No improvement in the frequency or duration of episodes was observed and therefore, treatment was discontinued. The dog with mild hypocobalaminemia (Case 4) was supplemented with cobalamin for 4 weeks until tested levels were normal.

### 3.6 Diet

At the time of the questionnaire, 4 dogs (4/5) were on commercial or home‐cooked gluten‐containing diets (GCD). The remainder dog (Case 2) was on a strict gluten free diet (GFD) since the age of 4 months because of gastrointestinal issues related to diet. His first episode occurred at 5 months old, while on GFD. He subsequently experienced 7 episodes within a year and according to the owners, the frequency of episodes decreased over time. Excluding that dog, a gluten‐free diet was trialed in 4 dogs (4/5). Follow up at 6 months from start of a strict gluten‐free diet trial revealed that all 4 dogs had experienced a noticeable reduction in episode frequency, duration, or a combination of both. That included Case 1 which had previously tested positive for gluten sensitivity. This dog was more challenging to manage for the owners because of recurrent scavenging episodes, experiencing episodes shortly after incidents of gluten‐containing‐diet ingestion.

### 3.7 Outcome

Owners were given 4 categories of progression to choose: (a) total remission of episodes; (b) partial remission (episode frequency was decreased or duration was shortened); (c) no remission (the episode frequency and duration was unchanged); or (d) worsened (episode frequency was increased or duration was prolonged). Although all dogs (5/5) experienced an episode within the previous year, every owner classified their dog as being in partial remission.

## 4 DISCUSSION

This study presents the clinical characterization of a novel PMD identified in WCS dogs. All 7 dogs exhibited episodes consistent with symmetric cerebellar ataxia, hypermetria, body swaying, head titubation, and wide‐based stance, in the absence of other PMDs such as episodes of hyperkinetic movements, dystonia or nerve/muscle hyperexcitability. Based on clinical signs, the term EA was deemed the most appropriate descriptive terminology. Thus, this descriptive study also reports the first documentation of EA in several dogs of the same breed; as previously, only 1 case report had described the presence of episodes of cerebellar ataxia in a 4‐year‐old Bichon Frise.^6^ This report was published 14 years ago, suggesting that EA is not commonly encountered in veterinary medicine. However, several factors could contribute to the under‐recognition of EA in dogs by clinicians, such as inadequate documentation of clinical findings or the absence of video footage. Similarly to the cases of this study, the previous report in a Bishon Frise involved a young dog at the onset of signs (4 months of age).^6^ Nevertheless, the breed and semiology of the episodes differed from those observed in our cases. The single case exhibited episodes characterized by stiffness in all 4 limbs, stiff gait with kyphosis, exaggerated jerky limb movements, frequent falling, reluctance to move, a wide‐based stance, and occasional discomfort.^6^ Additionally, the Bichon's episodes were preceded by vomiting and ptyalism, signs which were not observed in any of our cases.^6^ Recently, a group of Weimaraner dogs with an autosomal recessive paroxysmal dystonia‐ataxia syndrome was described. However, this syndrome differed from EA in that it presented as a combination of dystonia, ataxia, hypermetria, and intermittent anisocoria.^8^ In our cases, the absence of stiffness or other hyperkinetic movements (such as dystonia, dyskinesia, or nerve/muscle hyperexcitability) is a key distinguishing feature of WCS in this study, compared to other reports of movement disorders involving EA or swaying in dogs.

The phenomenology of the episodes was remarkably consistent across all cases examined in this study. Based on the constellation of signs—periods of cerebellar ataxia with body/truncal swaying and titubation, without associated tonic‐clonic or hyperkinetic movements, altered mentation, or autonomic signs—the episodes were classified as possible PMD.^1^ These episodes closely resembled previous reports of EAs in humans. In the human literature, PMDs are typically classified into 2 main groups: paroxysmal dyskinesias (PxDs) and EAs, depending on the predominant movement.^4^, ^9^, ^10^ Episodic ataxia is a well‐described syndrome characterized by transient recurrent episodes of cerebellar disfunction and truncal instability, varying in duration and frequency (from minutes to hours), with most patients exhibiting normal cerebellar function between episodes.^4^, ^5^, ^11^, ^12^ Episodic ataxia in humans might be accompanied by nystagmus, tremors, or myotonia.^5^, ^12^ Furthermore, episodes of incoordination could be associated with other clinical signs such as diplopia, dysarthria, nausea, dizziness, headache, cataplexy, distal weakness, malignant hyperthermia, sweating, hot flushes, palpitations, paroxysmal dyspnea, or sensory sings.^5^ Unfortunately, most of these clinical signs cannot be identified in dogs, as they are subjective manifestations of a disease apparent only to the patient. However, based on possible concomitant clinical signs, it can be hypothesized that signs of quietness (5/5), reduced response to calls (5/5), subdued behavior (1/5), and confusion during episodes, as well as dullness or lethargy (4/5) before episodes, could correspond to human symptoms such as dizziness or headache,^4^ which are difficult to characterize in dogs. Video recordings from 3 cases demonstrated distal limb weakness leading to or leaning over during episodes, while consciousness remained intact, as observed in Video S5. This presentation could be analogous to distal weakness observed in human medicine.^5^ Nevertheless, establishing a direct comparison between species remains challenging.

A characteristic sign present in all dogs was body/truncal swaying, which occurred when standing, sitting, or walking. This clinical sign has also been referred in veterinary literature as titubation, postural tremor,^13^ truncal ataxia,^14^ or truncal swaying.^15^ Body/truncal swaying refers to a wavering of the body from side to side, cranially and caudally, or occasionally dorsoventrally^14^ and it appears to be related to a cerebellar disfunction,^16^ specifically in the vermis or fastigial nucleus.^17^ Therefore body/truncal swaying has frequently been used to describe various cerebellar disorders in dogs. However, it should be noted that body/truncal swaying is not exclusive to cerebellar dysfunction^18^; it has also been used to describe the swaying during walks of an unstable trunk observed in dogs with spinal cord disorders.^17^ In these cases, the swaying is only side to side and is noted while walking^14^, ^17^ (Video S6). This must not be confused with head sway, which refers to the excursions of the head from side to side observed in bilateral vestibular lesions.^13^, ^16^ This body/truncal swaying has been also observed by the authors in dogs with paroxysmal dyskinesia or cerebellar diseases (Video S7).

In the human literature, both titubation and truncal ataxia are terms used in clinical neurology to describe distinct types of movement disorders. Although the literature is scarce, it seems that a distinction can be made between truncal ataxia/swaying and titubation (summarized in Table 1):

- Body/truncal ataxia or swaying refers to instability or lack of coordination in the trunk or axial muscles, leading to difficulty maintaining proper posture and balance while in standing or walking. Thus, truncal ataxia encompasses a range of symptoms related to impaired trunk control, including swaying, stumbling, and difficulty sitting or standing without support.^19^, ^20^, ^21^ It has been also referred as postural instability.^20^
- Titubation refers to a rhythmic, oscillatory movement of the head or trunk, typically occurring in standing or sitting posture (during attempts to maintain posture or balance). It manifests as rhythmical, high frequency (~3 Hz), small amplitude (5°‐10°) nodding or bobbing movements, therefore, this is usually referred as a head nodding or even as “stereotypic head movements.”^19^, ^22^ Although titubation is frequently described and classified as a tremor^22^, ^23^, ^24^ because it shares some similarities with tremors, it is not truly a tremor, as clinical differences have been identified based on literature descriptions: (a) Titubation affects only head or trunk,^19^, ^23^ while tremors can affect various body parts, including the hands, arms, legs, and head^25^, ^26^; (b) Tremors might be present at rest (resting tremor—not described in veterinary literature)^27^ or during voluntary movement (action tremor),^25^ while titubation typically occurs during attempts to maintain posture or balance.^20^, ^21^

**TABLE 1:** Summary of the key distinctions between body/truncal swaying and titubation.

Term	Semiology	Direction	Observed during	Physiopathology
Truncal swaying (AKA: truncal ataxia, body swaying, postural instability; Videos [Link], [Link], [Link], and S7)^19^	Impaired trunk control, encompassing swaying, stumbling, and difficulty sitting or standing without support^19^, ^20^, ^21^	From side to side, cranially and caudally, or occasionally dorsoventrally^19^, ^20^, ^21^	While in standing posture or walking^19^, ^20^, ^21^	Corresponds to the predominant involvement of the spinocerebellum^19^, ^20^, ^21^
Titubation (AKA: postural tremor, head nodding, stereotypic head movements; Videos S2 and S4)^19^, ^22^, ^23^, ^24^	Rhythmic, high frequency, small amplitude oscillatory movement of the head or trunk^19^, ^23^	From side to side and cranially and caudally^19^, ^20^, ^21^, ^22^, ^23^	While in a standing or sitting posture^19^, ^20^, ^21^, ^22^	Cerebellum. In humans also described in bilateral frontal lobe lesions due suspected damage to the frontopontocerebellar tract^25^, ^26^

Differentiating between titubation and body/truncal swaying can pose challenges, with the rhythmic nature of titubation frequently serving as the sole distinguishing feature from body/truncal swaying.^24^ It is important to note that while truncal swaying can be observed in both EA and PxDs, none of the WCS cases with EA exhibited stiffness or hyperkinetic movements, which are typically expected in dogs with PxDs (see Video S7). Additionally, titubation has neither been described nor observed by the authors in dogs with PxDs, whereas it was present in all WCS cases in this study. Besides titubation, body swaying and hypermetria were common findings in all cases, resembling previous reports of EA in humans. Therefore, the PMDs observed in our cohort of WCS were classified as EA.

Epileptic activity could be considered as an alternative etiology. Recently, suspected idiopathic vestibular epilepsy was reported in 10 dogs with pure vestibular signs, with interictal spikes noted in 3 cases.^28^ Similarly, a cerebellar link to seizure activity, termed cerebellar epilepsy, has been described in human medicine, but not in dogs.^29^ In cerebellar seizures, the episodes consist of focal or generalized epileptic seizures without the presence of cerebellar signs.^29^ On the other hand, epileptic seizures have also been associated with some forms of EA in humans, often with electroencephalography (EEG) abnormalities.^5^ Although seizures cannot be definitively excluded without EEG recordings during an episode, they were deemed unlikely here because of the prolonged signs and preserved consciousness despite generalized involvement and lack of improvement when antiepileptic drug was implemented in 1 dog. Furthermore, according to the classification by the International Veterinary Epilepsy Task Force consensus,^30^ the episodes observed in these WCS do not fit into any defined seizure types but could be considered preictal or postictal signs. Long‐term video‐EEG monitoring could be useful for further evaluating dogs with episodic signs. In human medicine, EA is frequently misdiagnosed as anxiety, seizures, or migraines before a correct diagnosis is established through genetic confirmation,^5^ highlighting the difficulty to achieve a diagnosis.

Initially, a cerebellar neuro‐localization was suspected based on the presence of hypermetria, ataxia, and titubation. However, because of generalized weakness and body swaying, a more diffuse central nervous system (CNS) involvement was also considered. Several differential diagnoses were explored, with metabolic diseases (eg, electrolyte imbalance, hypoglycemia, hepatic encephalopathy, cardiovascular disease) deemed most likely because of the episodic clinical signs and young age of the dogs. Structural or inflammatory CNS disorders were also evaluated. All cases underwent complete hemogram and biochemistry; bile acids and ammonia were tested in 2 and 3 cases, respectively. Other investigations, which varied among dogs, were generally normal. Intoxication was ruled out because of recurrent episodes in all cases and the absence of biochemical changes and other systemic signs. Although a cardiac disease^30^ or systemic hypertension^31^ could potentially explain intermittent weakness and ataxia despite normal auscultation findings, all cases involved athletic dogs with no exercise intolerance. Cardiac scans were performed in only 2 (2/7) dogs, with no Holter monitoring conducted. Ischemic transient attacks were deemed highly unlikely because of the young age of the dogs. For dogs with available follow‐up (5/7), ranging from 18 to 30 months from the first presentation, all remained physically and neurologically normal between episodes, making cardiovascular or structural/inflammatory brain diseases highly improbable. Furthermore, MRI and CSF analysis did not detect abnormalities when performed (3/7).

Therefore, after identifying 7 young dogs of the same breed (WCS) with identical signs and normal neurological interictal examinations, an inherited etiology for the episodes was considered the most likely differential diagnosis. However, further investigations are necessary. The etiology of EA in human medicine is classified in primary (genetic) or secondary (acquired).^4^, ^5^, ^9^ Primary EA in humans usually manifests in the first 2 decades of life, mainly during childhood or adolescence.^4^ Currently, 8 subtypes of EA are well‐described, with recent proposals for EA9 and EA10.^5^, ^9^ Episodic ataxia is common to all subtypes, but each subtype has a few clinical differences.^5^, ^9^ For primary EA, at least 5 genes and at least 8 loci involved have been identified. All identified genes, except for EA8 (UBR4), encode ion channel proteins and are important in excitatory neurotransmission.^4^, ^5^ Similarly, our WCS were very young (younger than 6 months) when the first episode occurred, and all dogs were bred in the UK, although in different regions. Pedigree status was not available for all dogs, and a common progenitor or lineage was not identified.

In our WCS, the episodes varied in duration from 30 minutes to 24 hours, typically lasting a few hours. The frequency ranged from 1 episode per week to 1 episode every 5 months. Additionally, some owners (3/5) reported that extraneous exercise and excitement occurring a day or several hours before the episodes were potential triggers. Similarly, in humans with EA, episodes can last from several minutes to hours and are often triggered by factors such as sudden movement, stress, exercise, caffeine, or alcohol.^5^

In human medicine, the diagnosis of primary EA is typically based on clinical features and the identification of specific gene mutations in the KCNA1, CACNA1A, or SCN2A genes.^5^, ^12^ However, this diagnosis can be challenging because of clinical variations, similarities with other disorders, and unusual presentations.^5^ Additionally, mutations in known EA genes account for less than half of patients with suspected EA, indicating genetic heterogeneity and the likelihood of undiscovered genes.^12^ To aid in diagnosis and rule out other potential comorbidities, various tests are employed. Brain imaging can evaluate structural brain lesions, EEG during a triggered attack can help differentiate EA from epileptic events (although baseline EEG abnormalities could be present in EA), and laboratory tests such as serum and CSF lactate or glucose can assist in diagnosing mitochondrial disorders. Specific blood tests can identify metabolic disorders and toxins related to secondary causes of EA.^5^

On the other hand, secondary causes of EA include vascular diseases (ischemic stroke, hemorrhage), inflammatory disorders (multiple sclerosis, postinfectious cerebellitis, Bickerstaff brainstem encephalitis, Miller‐Fisher syndrome), toxicity, peripheral vestibular disorders (eg, vestibular neuritis, labyrinthitis, Ménière's disease), paraneoplastic/autoimmune ataxia, primary neoplasia in the posterior fossa or cerebellum, and metabolic diseases (eg, maple syrup urine disease, pyruvate dehydrogenase deficiency, biotinidase deficiency, hypothyroidism). These conditions might be suspected by their onset after adolescence, negative family history, greater attack variability, and typically abnormal laboratory and imaging findings.^5^ None of the investigations in our cohort revealed abnormal findings; however, the lack of uniformity in performed tests is a study limitation. Despite various hypotheses, the etiology of EA in our cases remains unknown. The young age of onset, breed uniformity, and absence of other abnormalities suggest a possible inherited primary cause.

Information about the progression was available in 5 dogs, with follow‐up periods ranging from 18 to 30 months. A gradual reduction in episode frequency was observed over time. In 4 dogs where GFD was initiated, a reduction in the frequency of episodes was seen. In recent years, a relationship between PMDs and gluten sensitivity has been reported in human medicine^32^ and in dogs.^33^, ^34^, ^35^ In fact, a clinical response to GFD has been observed in human patients with ataxia. This clinicopathological entity is termed gluten ataxia and is defined as idiopathic sporadic ataxia with serologic evidence of gluten sensitivity (positive antigliadin antibodies) in the absence of an alternative cause. In these cases, the presence of enteropathy (celiac disease) is not a prerequisite for the diagnosis of gluten ataxia.^36^ This potential link warrants further investigation. Only 2 cases in our study were tested for antigliadin antibodies; one of these tested positive and showed an improvement after initiating a GFD. Despite that, it is important to note that any relationship between diet and clinical response should be reported with caution, considering the possibility of spontaneous remission, as documented in previous studies on PxDs in dogs.^37^ In our cases, 2 dogs (2/5) experienced relapses shortly (24‐48 hours) after the accidental ingestion of gluten‐containing food. However, in 1 dog with a relapse linked to diet, the owners also reported a temporary increase in episode frequency that coincided with the arrival of a newborn in the household while the GFD was discontinued. Furthermore, the remaining dog that was already on a GFD suffered episodes despite the diet, though their frequency had decreased. This highlights that multiple factors might occur at the same time and the difficulty to demonstrate a significant correlation in studies with a small number of cases.

The outcome of our cases with EA contrasts with the previous case report of a 4‐year‐old Bichon Frise. The Bichon Frise experienced a gradual decline over several years, with complete resolution of episodes only achieved following treatment with 4‐aminopyridine, a potassium channel blocker.^6^ Other medications, including prednisone, phenobarbital, and acetazolamide, proved ineffective, and a GFD trial was not attempted.^6^ In contrast, in humans with primary EA, symptoms typically begin in childhood or adolescence and tend to improve or resolve with age, with a normal life expectancy.^9^ The number of attacks generally decreases with age, suggesting the presence of compensatory mechanisms in the brain.^4^

Our study had several limitations, including a small sample size, a retrospective character and non‐standardized diagnostic protocols. A complete diagnostic evaluation was not uniformly performed across all dogs, and more specific tests (such as EEG or thrombo‐elastography) were not conducted, thereby limiting the ability to accurately rule out secondary etiologies of EA. In addition, a detailed pedigree was only available for 1 case. Despite these limitations, the cases of this study provide an initial yet important description of a new PMD in dogs.

In conclusion, this study characterizes the clinical manifestation of a novel PMD, termed EA in the WCS breed. All 7 dogs exhibited symmetric cerebellar ataxia, hypermetria, titubation, body/truncal swaying, and wide‐based stance. Findings have been cautiously compared with human EA descriptions, and some characteristics differ. The underlying mechanism of EA remains unknown, and its diagnosis is presumptive in these cases. Prognosis appears favorable, with a decreased episode occurrence over time. A GFD apparently reduced episode frequency and therefore, it is worth considering implementing a GFD in dogs with suspected primary EA. The study provides a foundational database for future research, including genetic studies, gluten sensitivity and long‐term clinical monitoring.

## CONFLICT OF INTEREST DECLARATION

Authors declare no conflict of interest.

## OFF‐LABEL ANTIMICROBIAL DECLARATION

Authors declare no off‐label use of antimicrobials.

## INSTITUTIONAL ANIMAL CARE AND USE COMMITTEE (IACUC) OR OTHER APPROVAL DECLARATION

Approved by the Research Ethics Committee of the School of Veterinary Medicine of the University of Glasgow (EA42/22).

## HUMAN ETHICS APPROVAL DECLARATION

Authors declare human ethics approval was not needed for this study.

## Supporting information


**Data S1:** Supporting Information.

**VIDEO S1:** Video footage of 3 WCS during an episode of EA. Note features of cerebellar ataxia, hypermetria and a wide base stance with alert mentation.

**VIDEO S2:** Video features of 2 WCS with head titubation while in a sitting posture. Note the characteristic rhythmic, fine, oscillatory movements of the head.

**VIDEO S3:** Three WCS exhibited characteristic signs of body/truncal swaying. In the second WCS, body/truncal swaying was observed in both directions separately: From side to side and cranially and caudally.

**VIDEO S4:** A WCS showing a combination of mild truncal/body swaying in cranially and caudally direction with head titubation while in standing position. Mild wide base stance in thoracic limbs is noted.

**VIDEO S5:** Distal limb weakness causing the dog to lean down or almost collapse during an episode of EA with preserved consciousness in 3 WCS.

**VIDEO S6:** Example of truncal swaying (in lateral direction) and proprioceptive ataxia in a German Shepard dog with cervical spondylomyelopathy.

**VIDEO S7:** Truncal/body swaying in dogs with different diseases: A Chihuahua with paroxysmal dyskinesia (characterized by intermittent and irregular flexion of both hind limbs), and a Yorkshire Terrier with cerebellar disease (characterized by cerebellar ataxia and titubation).
