# Supplementary material for: Clinical characterization of a novel episodic ataxia in young working Cocker Spaniels
Source: J Vet Intern Med. 2024 Dec 23;39(1):e17268. doi: 10.1111/jvim.17268 (PMC11665963; doi:10.1111/jvim.17268)
Supplement: Supplementary file 1 — Data S1: Supporting Information. [file JVIM-39-e17268-s002.docx]

**S1.** **Materials and Methods**- Table including signalment, episode characterization, investigations, and response to GFD of the affected dogs.

| **Case** | **Signalment** | **Onset** | **Length, frequency and triggers** | **Phenomenology from video footage** | **Investigations** | **Diet before consultation** | **Gluten free diet trial** | **Response to diet trial** |
| --- | --- | --- | --- | --- | --- | --- | --- | --- |
| ***Case 1*** | 3 yo FE | 3 mo | **Duration:** 30 min to 12 h  **Frequency:** 4 per month  **Trigger:** Exercise, excitement | **Posture:** Normal.  **Gait:** cerebellar ataxia of all limbs, hypermetria, wide base stance. Collapse.  **Other signs:** Body and head titubation. | CA, CBC, Hem, NA | GCD, scavenger | Yes | Decreased frequency of episodes. Experiences increased frequency of episodes when diet is altered. |
| ***Case 2*** | 3yo ME | 5 mo | **Duration:** hours  **Frequency:** one every 2 months  **Trigger:** Exercise | **Posture:** Kyphosis.  **Gait:** cerebellar ataxia of all limbs, hypermetria, wide base stance. Collapse.  **Other signs:** Body and head titubation. Urination during initial episodes. | AGA IgG, BAST, CA, CBC, CSF, FA, Fo and B12, Hem, iCa, L-car, MRI, NA, NH3, *Tox and Neo*, UA, UOA | GFD | Already GFD | Decreased frequency of episodes. |
| ***Case 3*** | 3yo FN | 5 mo | **Duration:** up to 24h  **Frequency:** one every month  **Trigger:** Exercise, excitement | **Posture:** Low lumbar carriage.  **Gait:** cerebellar ataxia of all limbs, hypermetria, wide base stance. Collapse.  **Other signs:** Body and head titubation. Generalized, intermittent fine tremors as well as intention tremors. | CBC, Hem, NA | GCD | Yes | Decreased frequency of episodes. Experienced increased frequency of episodes when diet was altered and when a newborn was brought into the household (potential stressor). |
| ***Case 4*** | 1yo FE | 4 mo | **Duration:** 1-3h  **Frequency:** 2 in total  **Trigger:** unknown | **Posture:** Kyphosis.  **Gait:** cerebellar ataxia of all limbs, hypermetria.  **Other signs:** Body and head titubation. | BAST, CBC, Hem, NA, *Tox and Neo*, UA, UOA | GCD, scavenger | No | N/A |
| ***Case 5*** | 3yo FN | 4 mo | **Duration:** 1-2h.  **Frequency:** one every 5 months  **Trigger**: Exercise, excitement. | **Posture:** Kyphosis.  **Gait:** not able to evaluate.  **Other signs:** Body and head titubation. Urination during initial episodes. | CBC, Hem, NA, NH3, *Tox & Neo,* VBG | GCD, scavenger | Yes | Decreased frequency of episodes. |
| ***Case 6*** | 4yo FE | 4 mo | **Duration:** seconds to minutes  **Frequency:** 4 in one month  **Trigger:** Unknown | **Posture:** Wide base stance.  **Gait:** cerebellar ataxia in the front limbs.  **Other signs:** Body and head titubation. | AGA IgG, CBC, CSF, Fo and B12, Hem, iCa, L-car, MRI, NE, NH3, UA, UOA | GCD | No | N/A |
| ***Case 7*** | 3yo ME | 3 mo | **Duration:** 1-2h  **Frequency:** daily  **Trigger:** unknown | **Posture:** Normal.  **Gait:** cerebellar ataxia in all four limbs.  **Other signs:** Body and head titubation. Collapse. | CBC, CK, CRP, CSF, cTnl, FA, Hem, Ca, MRI, NE, NH3, UA, UOA | N/A | No | N/A |

**Abbreviations:** AGA IgG, gliadin IgG and transglutaminase-2 IgA; BAST, Bile Acid Stimulation Test; CA, cardiac assessment; CBC, complete blood count; CK, Creatinine kinase; CRP, C-reactive protein; CSF, cerebrospinal fluid; cTnl, troponin; FA, fructosamine; Fo & B12, folate and cobalamin; GCD, Gluten-Containing Diet, GFD, Gluten-Free Diet; Hem, Hematology; iCa, ionised calcium; L-car, levocarnitine; MRI, magnetic resonance imaging; N/A, not applicable; NE, neurological examination; Neo., *Neospora caninum* serology; NH3, ammonia; Tox., *Toxoplasma gondii* serology; UA, urinalysis; UOA, urine organic acids; VBG, venous blood gases.

**S2 Materials and Methods –** Questionnaire for clients with WCS dogs with suspected EA

**SECTION ONE – Your pet’s information**

- Pet name:
- Date of birth and age:
- Breed (Kennel Club name):
- Sex:
- Last weight taken (approximate date):
- Entire/Neutered:
- Date of neutering:
- Diet:
- Exercise (walks per day/week, length of walks):
- Where did you get your dog from? Please specify if breeder, family friend etc, location:
- Does your dog hold a pedigree? If so, could you include a copy of the pedigree as it could help to track if there is a line of affected dogs in her/his pedigree line.
- Do you know if the dam, sire or other litter mates are affected by the same disorder?

□ Yes □ No

- Please state your current primary veterinarian (please, state the same practice as above if same as previously mentioned).

**SECTION TWO - Your pet’s condition**

- At what age did your dog start showing signs? (weeks, months, years...) If you cannot remember the precise age or date when your dog had the first episode, would you be able to tell us whether your dog was:
  - - <6mo
    - 6-12mo
    - <12mo-1.5yo
    - <1 yo-2yo
    - <2yo
- Date of last known episode:
- Are there any signs of “aura” meaning, a period of altered behavior in which a dog may hide, appear nervous, or seek out the owner before having an episode?

□ Yes □ No

- Please describe the episodes in terms of what your dog is doing:
- What would you consider as the primary/dominant sign?
- Are episodes similar/identical? If not, please specify what differs.

□ Yes □ No

- What is the average length of an episode (describe):

□ <5 mins □ 5-10 mins □ 10-30 mins □ >30 mins

- How often do episodes occur? (every day, every week, every month, every 3 months etc.).
- In case of daily episodes, what is the frequency:

□ 1 time □ 2 times □ 3 times □ 4 times □ <4 times □ Random

- Would you describe the signs as being *focal* (one part of the body, e.g. face) *segmental* (affects two or more parts of the body that are adjacent or close to one another. For example: eyelids and mouth, eyelids and vocal cords, neck and trunk, neck and upper limbs), *lateralized* (to either side right/left of the body), *generalized* (affecting the entire body).
- Focal
- Segmental
- Lateralized
- Generalized
- Would you describe your dog as being conscious or unconscious (becomes unconscious/unaware of where he/she is and who you are) during the episode?
- Can you get the dogs’ attention during the episode (calling/touching):
- Yes
- No
- Does your dog seem to be scared/anxious during episodes?
- Yes
- No
- Is your dog to be in pain during episodes?
- Yes
- No
- Does your dog exhibit trouble walking during the episode:
- Yes (describe):
- No
- Does your dog do any of the following during an episode (please select all that apply)?
  - Reluctant to move or stand
  - Your dog will still attempt to walk
  - Become recumbent (lie down)
  - Rhythmic body/leg shaking/jerking movements
  - Twisting of the body and fine tremors of the head
  - Twitches of his/her head muscles only
  - Swaying of the head/body
  - Head bobbing
  - Makes chewing movements
  - Back limbs are often affected to a greater degree than the front limbs
  - Salivates (drools) more than usual
  - Urinates (pee) with no awareness of doing it
  - Defecates (poo) with no awareness of doing it
  - Vomits (poo) with no awareness of doing it
  - Normal walking
  - Stiff walking
  - Drunker walking
  - Normal posture
  - Arched posture
  - Restlessness
  - His/her eyes are moving randomly during episodes (nystagmus)
  - Aggressiveness
  - Head tilt
  - Other, (please specify):
- Once the episode has passed, in the minutes/hours following an episode, does your dog show any of the following (please select all that apply):
  - Disorientation
  - Aggressive behavior
  - Acting fearful
  - Restlessness
  - Pacing (walking without a clear purpose)
  - Lethargy
  - Deep sleep
  - Hunger
  - Thirst
  - Wobbly when walking Blindness (bumping into things)
  - Rumbling sounds of the tommy, more defecation than usual
  - No signs at all, he/she goes back to normal
  - Other (please specify):
- Is your dog completely normal during the days between episodes?
- Yes
- No
- What the dog is doing when episodes start?
- Episodes happen during rest/sleep
- Exercising (playing, walking, etc.)
- They may happen either during rest/sleep and during activity
- Which time of the day are the episodes more frequent:

□ Morning (6am-12am) □ Afternoon (12am-5pm) □ Evening (5pm-8pm)

□ Night (8pm-6am) □ Random

- Would you say there is any triggers? (any external or internal stimuli that could start the episodes and that could include sleeping or resting, an abrupt movement, standing up, standing sown, exercise, light, sound, startle, stress/anxiety, emotional stress, etc.)
- Have you have recognized any exacerbating factors (typical aggravating factors include a concurrent condition/disease, a bitch in heat, a new treatment drug, a new diet etc.).
- According to you, how would you describe the evolution of signs?
  - My dog has deteriorated (episodes occur more often)
  - My dog has been stable (same frequency, severity and duration of episodes)
  - My dog has improved (episodes occur less often)
  - My dog has gone into remission (I have not witnessed any more episodes). If this is the case, please specify approximately the last time your dog suffered an episode.
- Can you predict when your dog is about to have an episode? Why?
- Yes
- No
- Diet the dog was taking when episodes occurred (specify type and brand):
- Diet the dog is currently taking (specify type and brand):
- Have you have noted your dog having barking/voice issues or changes? (Describe)
- Yes
- No

**SECTION THREE - Previous investigations performed in your pet**

- If you are aware, which tests have been performed by your vet to identify the cause (please tick all appropriate boxes)?
  - - No test has ever been performed
    - I am not sure if and which tests have been performed
    - Physical examination
    - Neurologic examination
    - Blood tests
    - Urine test
    - Computed tomography (CT) of the brain
    - Magnetic resonance imaging (MRI) of the brain
    - Cerebrospinal fluid analysis
    - ECG
    - Cardiac exam
    - Other (please specify):
- Where any of these test results NOT normal, if yes specify:

**SECTION FOUR - Treatment**

- Has your dog been specifically put on treatment to alleviate the frequency of these episodes? If yes, please select below:
  - Phenobarbitone (Epiphen or Phenoleptil)
  - Potassium bromide (Libromide or Epilease)
  - Imepitoin (Pexion)
  - Levetiracetam (Keppra)
  - Gabapentin (Neurontin)
  - Zonisamide (Zonegran)
  - Pregabalin (Lyrica)
  - Other (please specify):
- If your dog is on any medication, please specify the dose (in milligrams or similar international unit) and frequency (once a day, twice a day...)
- When did your dog start taking medication? Please provide us with a date.
- Has your dog improved since starting medication?
  - Yes, my dog suffers from less episodes in comparison to when she/he was not on treatment
  - No, the frequency of episodes has not changed since starting treatment
- Please tell us anything else about your dog's movement disorder that you think we should know.

**SECTION FIVE - Comorbidities**

Was the dog suffering from any other condition while the episodes started?

□ Hypersensitivity (allergic) skin condition □ Gastrointestinal problems

□ Traumatic event □ Other:

- Has your dog been diagnosed with any other condition?
- Yes - which condition?
- No
- If yes, is your dog on treatment for it? Please specify what medication your dog is on, the dose (in milligrams, milliliters or similar international unit) and frequency (once a day, twice a day...).
- When was your dog first diagnosed?
- If possible, could you please provide video footage of one or more of the episodes of your dog

Please feel free to add any additional comments:
